# Extent of resection and its association with overall survival in newly diagnosed IDH wildtype glioblastoma treated with concomitant radiochemotherapy: a systematic review and meta-analysis

**DOI:** 10.1016/j.bas.2025.105867

**Published:** 2025-11-21

**Authors:** Wietse Geens, Gzim Rizani, Nicole Del Gaudio, Félix Buyck, Frederick Van Gestel, Michaël Bruneau, Bart Neyns, Johnny Duerinck

**Affiliations:** a Department of Neurosurgery, UZ Brussel, Laarbeeklaan 101, 1090, Brussels, Belgium; b Vrije Universiteit Brussel, Faculty of Medicine, Laarbeeklaan 103, 1090, Brussels, Belgium; c Department of Medical Oncology, UZ Brussel, Laarbeeklaan 101, 1090, Brussels, Belgium

**Keywords:** Extent of resection, Prognosis, Newly diagnosed IDHwt glioblastoma, Systematic review, Meta-analysis

## Abstract

**Background:**

Extent of resection (EOR) is a well-known prognostic factor in patients with newly diagnosed IDH-wildtype glioblastoma. However, reported survival times across resection categories vary between reports, and outcomes of submaximal or supramaximal resection remain less well defined.

**Methods:**

We conducted a systematic review and meta-analysis on the association between EOR and overall survival (OS) in patients with newly diagnosed IDH-wildtype glioblastoma treated with chemoradiotherapy. Studies were included if OS was reported by EOR category. Risk ratios (RRs) for 1- and 2-year survival were pooled using a random-effects model. Study quality was assessed using the Newcastle-Ottawa Scale.

**Results:**

Thirty-one studies involving 26,167 patients were included. Supramaximal resection (SupraMR) was associated with significantly improved 2-year survival compared to maximal CE resection (MR) (RR 0.70, 95 % CI 0.55–0.88). Compared to submaximal resection (subMR), MR was associated with higher 1-year survival (RR 0.59, 95 % CI 0.53–0.67) and 2-year survival (RR 0.82, 95 % CI 0.77–0.87). Biopsy alone was associated with the poorest outcome. Findings remained robust in sensitivity analyses excluding SEER and RTOG cohorts.

**Conclusions:**

Increasing EOR seems to be associated with improved survival in newly diagnosed IDH-wildtype glioblastoma. SupraMR offers the greatest benefit, while submaximal resection appears to be more favorable than biopsy. These findings support the prognostic relevance of EOR and underscore the need for prospective studies with standardized resection classifications. The balanced summary of survival data for each resection class provided in this review can serve as a basis for effect estimation and sample size calculations in future trials.

## Highlights

• Largest meta-analysis (n = 26,167) to date assessing EOR in IDH-wt glioblastoma treated per the EORTC 26981/22981 protocol.
• Increasing EOR seems to have significantly better survival: SupraMR resection > maximal CE resection > subMR > biopsy.
• Supramaximal resection (SupraMR) improves 2-year survival compared to maximal CE resection, with median OS of 28.2 months vs 17.5 months.
• Even submaximal resection offers better survival than biopsy, but the benefit is modest and potentially confounded.
• Study highlights urgent need for prospective trials using standardized resection definitions (e.g., RANO-Resect).

## 1 Introduction

Glioblastoma (GBM) is the most common and aggressive primary malignant brain tumor in adults, known for its infiltrative nature and poor prognosis. Amid ongoing efforts to define the optimal therapy, the current standard of care for newly diagnosed GBM, established in 2005, involves maximal safe surgical resection followed by concurrent and adjuvant temozolomide chemotherapy with radiotherapy (Stupp et al., 2005). The surgical component can range from a minimally invasive diagnostic biopsy to a craniotomy aimed at maximal CE resection or even supramaximal resection (SupraMR). Techniques such as 5-aminolevulinic acid (5-ALA)-guided or intraoperative MRI have significantly enhanced the potential for achieving a greater extent of resection (EOR) (Picart et al., 2024; Coburger and Wirtz, 2019). However, the pursuit of maximal tumor removal must be carefully balanced against the risk of new or worsened postoperative neurological deficits, which are recognized as independent negative prognostic factors (Picart et al., 2024; Roder et al., 2023; Rahman et al., 2017). Despite technological and surgical advancements, achieving significant improvements in overall survival (OS) remains a challenge.

Numerous large retrospective studies have suggested that increased EOR in patients with newly diagnosed GBM is associated with improved survival. Earlier retrospective data indicated incremental survival benefits for EORs starting from 78 % to 98 % (Sanai et al., 2011; Brown et al., 2016). However, a significant limitation of many of these older studies and reviews is the inclusion of both IDH-wildtype and IDH-mutant tumors, which were not yet distinctly classified prior to the 2021 World Health Organization (WHO) reclassification (Louis et al., 2021). Moreover, more recent and rigorous reports have indicated that absolute residual volume of tumor remaining is a stronger prognosticator than relative EOR (Karschnia et al., 2022; Gerritsen et al., 2021, 2023).

Persistent debate and considerable variability in practice remain within this field. While increased extent of resection (EOR) is associated with improved survival up to the point of maximal CE resection (MR), the additional benefit of SupraMR over MR remains uncertain. Likewise, it is unclear whether submaximal resection of glioblastoma confers any survival advantage compared to biopsy alone. Resolving these questions will require prospective, preferably randomized, clinical trials (Duerinck et al., 2024). Accurate survival estimates for each resection category are essential for designing such studies, including effect size estimation and sample size calculations. Therefore, we conducted a systematic review and meta-analysis to generate comprehensive survival data stratified by resection class, providing a foundation for future trial planning.

## 2 Methods

This systematic review was conducted according to the PRISMA 2020 guidelines (Page et al., 2021) and complies with relevant standards. The recommendations of the Cochrane Collaboration were followed.

A comprehensive systematic literature search was conducted across major electronic databases, including PubMed, Web of Science, and Embase, to identify relevant studies published from 2005 to October 2024, starting with the landmark publication of the concomitant radiochemotherapy paper by Stupp et al. (2005). The search strategy combined relevant keywords and MeSH terms related to “glioblastoma,” “IDH-wildtype,” “extent of resection” (including “gross total resection,” “subtotal resection,” “biopsy,” and related terms), and “survival” (including “overall survival”). The search was refined to include only studies involving human subjects. Additionally, reference lists of relevant review articles and meta-analyses were manually screened to identify any potentially missed studies. Expert consultation was also considered to ensure the inclusion of all relevant literature.

Studies were included based on the following eligibility criteria: (1) the study was a published randomized controlled trial, prospective non-randomised study or a retrospective cohort study concerning newly diagnosed, histopathologically confirmed IDH-wildtype GBM, in which EOR and survival were explicitly investigated as a parameter and mentioned in the abstract; (2) the study population consisted of adult patients with at least 90 % of patients with histopathologically confirmed IDH-wildtype GBM; (3) the intervention involved surgical excision (with or without biopsy subgroup) for newly diagnosed GBM; (4) the study reported 1 and 2 year survival rates stratified by EOR, or provided sufficient data to allow extraction of those from Kaplan-Meier (KM) curves using appropriate software (PlotDigitizer, 3.1.6, 2025, https://plotdigitizer.com) (5) the study included a comparison between at least two groups with a different EOR.

Studies were excluded based on the following criteria: (1) systematic reviews, technical notes, letters to the editor, and comments; (2) studies not written in English and no English translation available; (3) studies focusing on recurrent or multiple GBM diagnoses in the same patients; (4) studies that included a mixed population with other brain tumor types, unless data specific to IDH-wildtype GBM could be extracted separately; (5) studies with less than 90 % of the included GBM cases histopathologically confirmed as IDH-wildtype; (6) the total study population comprised fewer than 100 patients; (7) conference papers or studies where the full text was not available; (8) studies where survival outcomes were not reported, nor could they be extrapolated from KM curves; (9) studies that did not provide a comparison between different extent of resection groups (SupraMR, MR, subMR, biopsy); (10) studies conducted prior to the reporting of the EORTC 26981/22981-NCIC CE3 study results in 2005 or in which fewer than 85 % of patients received standard-of-care treatment following surgery that was defined by this study and consists of concomitant and adjuvant radiochemotherapy with temozolomide (further referred to as ‘standard radiochemotherapy’); and (11) studies focusing solely on pediatric or elderly patients.

Two reviewers (W.G. and G.R.) independently screened the titles and abstracts of all retrieved records, and articles meeting our inclusion criteria were subjected to a full-text review. From each eligible study, key characteristics were extracted, including author, publication year, study design, and sample size. Recorded data included EOR according to the RANO classification (SupraMR, MR (complete CE resection and near total CE resection), subMR (subtotal and partial resection combined), or biopsy, as defined in the original study) and primary outcome measures: median overall survival (mOS), 1-year survival rate, 2-year survival rate, and hazard ratios (HR) with corresponding 95 % confidence intervals (CI) (Karschnia et al., 2022). In cases where survival metrics were not explicitly stated, Kaplan–Meier curves were digitized and analyzed using established methods to estimate median overall survival and 1- and 2-year survival rates. Any discrepancies that arose during screening or data extraction were resolved through iterative discussions until a consensus was reached.

### 2.1 Quality scoring

The Newcastle-Ottawa Scale was used to assess the quality of the included studies. High-quality studies were defined based on the following: (1) study design, (2) defined inclusion and exclusion criteria, (3) adequate length of follow-up, (4) detailed information about tumor molecular analysis, (5) detailed and separate information about IDH mutated (if present) and IDH wildtype outcomes, and (6) comprehensive treatment-related data. A star rating of 0–9 was allocated to each study. The quality assessment was performed independently by two authors, and a third author (J.D.) resolved any discrepancies. Studies receiving six or more stars are considered high-quality. The final scoring per article can be found in Supplementary Table 1.

### 2.2 Meta-analysis

Relative risks (RRs) and 95 % CIs for each of our comparisons of interest and HR with 95 % CI were calculated using the random effects model in Review Manager Web (Version 9.0.0; Cochrane Collaboration [https://www.cochrane.org]). The random effects model was used instead of the fixed effects model because of the heterogeneity among the studies, to provide a more conservative and clinically reliable interpretation of the summarized statistics and 95 % CIs.

Heterogeneity between studies was assessed using the I^2^ statistic, with values of 25 %, 50 %, and 75 % indicating low, moderate, and high heterogeneity, respectively. Sensitivity analyses were performed in case of outliers or to identify studies attributing to high heterogeneity. We repeated each meta-analysis after excluding Surveillance, Epidemiology, and End Results (SEER) data, and again after excluding both SEER and Radiation Therapy Oncology Group (RTOG) data, to assess the effect of the large sample sizes in those studies on the overall meta-analytic estimates and heterogeneity. Significance was established using CIs at a 95 % level or P < .05. Publication bias was assessed using funnel plots. This methodological approach aimed to provide a rigorous and comprehensive synthesis of the evidence regarding the prognostic role of the EOR in newly diagnosed IDH-wildtype glioblastoma patients treated with standard radiochemotherapy.

## 3 Results

### 3.1 Literature search

**Fig. 1:**
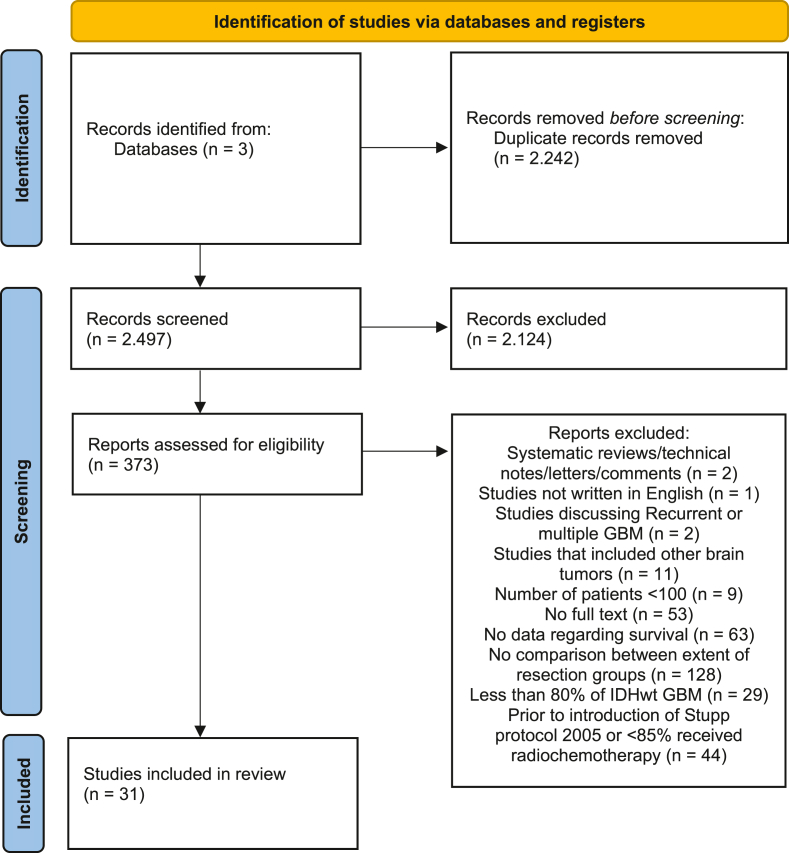
Preferred Reporting Items for Systematic Reviews and Meta-Analyses flow diagram.

An exhaustive systematic literature search was conducted across three distinct electronic databases, initially retrieving 4739 records. After duplicates removal, 2497 unique citations remained. During the initial screening phase, 2124 articles were excluded based on predefined inclusion and exclusion criteria, resulting in a final selection of 373 articles for detailed full-text assessment. After a full-text review, 31 papers were retained and included in at least one comparison in the subsequent meta-analysis, representing a total of 26,167 unique patients (Picart et al., 2024; Roder et al., 2023; Karschnia et al., 2022; Gerritsen et al., 2023; Aabedi et al., 2022; Adeberg et al., 2016; Ahmadipour et al., 2019; Amelot et al., 2017; Capellades et al., 2018; Castro et al., 2024; Hall et al., 2019; Hallaert et al., 2020; Byun et al., 2019; Marchi et al., 2019; Potharaju et al., 2018; Salvati et al., 2020; Valente Aguiar et al., 2021; Villanueva-Meyer et al., 2017; Padwal et al., 2016; Park et al., 2023; Mendoza Mireles et al., 2023; Kreth et al., 2013; Jiang et al., 2017; Yang et al., 2022; Drexler et al., 2023; Tropeano et al., 2024; Di et al., 2023; Pessina et al., 2017; Yoo et al., 2022; Molinaro et al., 2020; Kim et al., 2019). The study selection process is illustrated in the PRISMA flowchart (Fig. 1).

### 3.2 Body of evidence quality (GRADE rating)

Our assessment of the quality of evidence using the GRADE criteria was low for all overall survival outcome measures.

### 3.3 Meta-analysis for overall survival at 1 year

**Fig. 2:**
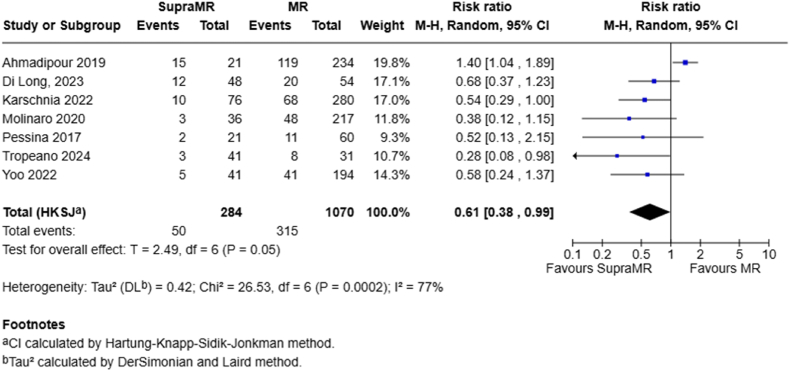
Forest plots depict RRs at 1 year for SupraMR vs MR.

Seven studies compared SupraMR to MR in terms of one-year outcomes. Under a random-effects model, the pooled RR was 0.61 (95 % CI 0.38–0.99; P = .05), indicating a statistically significant survival benefit for SupraMR (Fig. 2). However, due to outlier results from the study by Ahmadipour et al. (which favored MR over SupraMR), a sensitivity analysis was performed. When this study was excluded, the pooled RR improved from 0.61 (95 % CI 0.38–0.99; P = .05, I^2^ = 77 %) to 0.54 (95 % CI 0.41–0.71; P = .002; I^2^ = 0 %). In other words, this single outlier significantly impacted both the estimated effect of SupraMR at one year (RR increase from 0.54 to 0.61) and was responsible for all statistical heterogeneity (I^2^ from 0 % to 77 %). This suggested that the Ahmadipour study, which reported a point estimate in favor of MR, was the primary contributor to heterogeneity and diminished the overall observed effect (Supplementary Fig. 1).

**Fig. 3:**
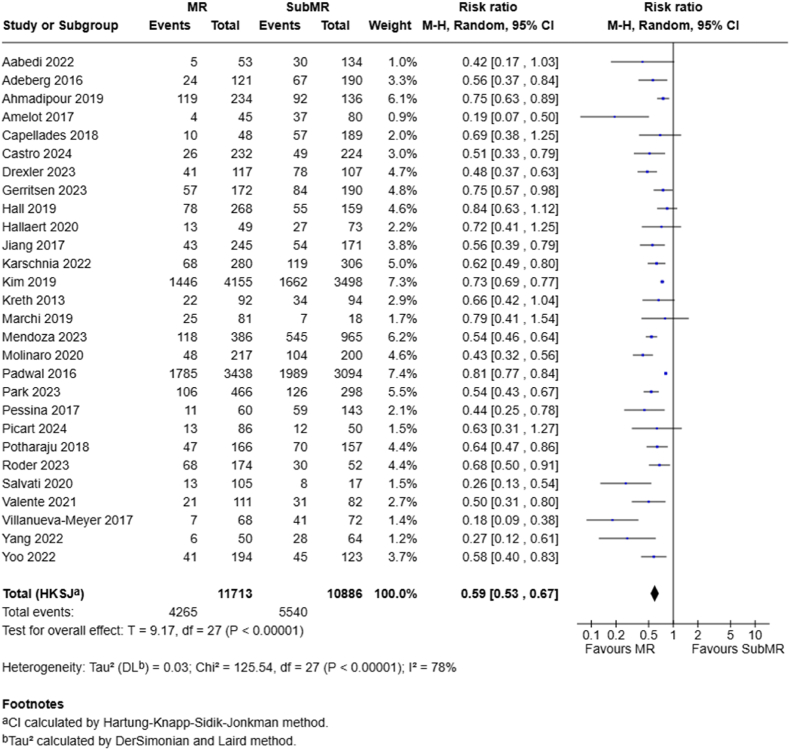
Forest plots depict RRs at 1 year for MR vs subMR.

For the comparison of MR vs subMR at 1-year OS, 28 studies were included in this analysis. The overall RR at 1 year is 0.59 (95 % CI, 0.53–0.67; P < .00001), significantly favoring MR over subMR (Fig. 3).

**Fig. 4:**
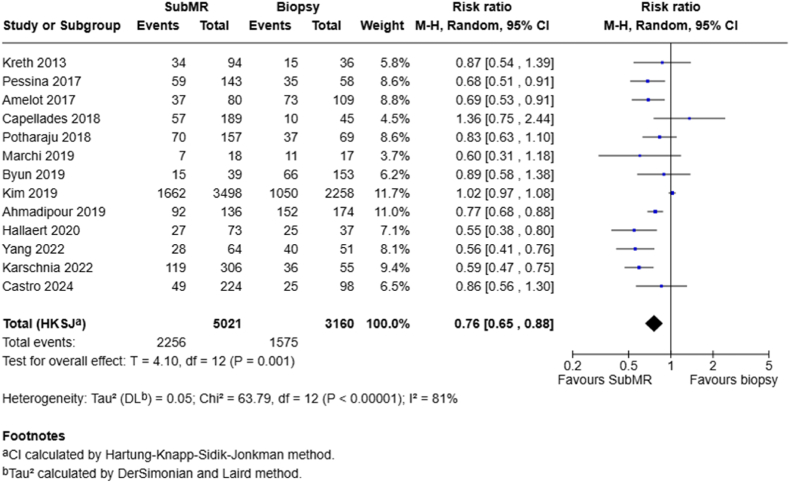
Forest plots depict RRs at 1 year for subMR vs biopsy.

Thirteen studies compared subMR with biopsy in terms of one-year outcomes. Under a random-effects model, the pooled RR was 0.76 (95 % CI 0.65–0.88; P = .001), indicating a significant 24 % reduction in one-year risk with subMR (Fig. 4).

### 3.4 Meta-analysis for overall survival at 2 years

Seven studies compared two-year survival after SupraMR versus MR. Under a random-effects model, the pooled RR was 0.70 (95 % CI, 0.55–0.88; P = .009), indicating a 30 % relative reduction in two-year mortality with SupraMR compared to MR (Supplementary Fig. 2). These findings suggest that, on average, extending resection beyond contrast-enhancing margins confers a durable survival benefit at the two-year mark. Sensitivity analysis without the Amadipour study is shown in Supplementary Fig. 3.

Across 28 studies, two-year OS was significantly higher following MR compared with subMR. Under a random-effects model, the pooled RR was 0.82 (95 % CI 0.77–0.87; P < .00001), indicating an 18 % relative reduction in two-year mortality for MR versus subMR (Supplementary Fig. 4).

Across 13 studies comparing subMR with biopsy alone, two-year mortality was modestly lower in the subMR group. The random-effects pooled RR was 0.91 (95 % CI 0.85–0.98; P = .02), indicating an 8 % relative reduction in two-year mortality for subMR versus biopsy alone (Supplementary Fig. 5).

### 3.5 Pooled mOS and OS ratios at 1 and 2 years

To translate our findings into clinically actionable metrics, we quantified and compared pooled mOS and pooled OS at one and two years for each surgical category: SupraMR, MR, subMR, and biopsy alone. This approach enables clinicians to readily apply these numbers when counseling patients on the expected short- and intermediate-term benefits of more extensive resection compared to limited or non-resectional management (Table 1).

**Table 1:** Pooled mOS and OS ratios at 1 and 2 years for the corresponding extent of resections.

Extent of Resection	No. of Studies	Total N	mOS (months)	1-Year OS, %	2-Year OS, %
Supramaximal resection	7	284	28.2	82.4 %	51.8 %
Maximal CE resection	30	11798	17.5	63.6 %	29.1 %
Submaximal resection	29	10925	12.5	49.1 %	18.9 %
Biopsy alone	13	3160	12.2	50.2 %	21.7 %

### 3.6 Hazard ratios meta-analysis

**Fig. 5:**
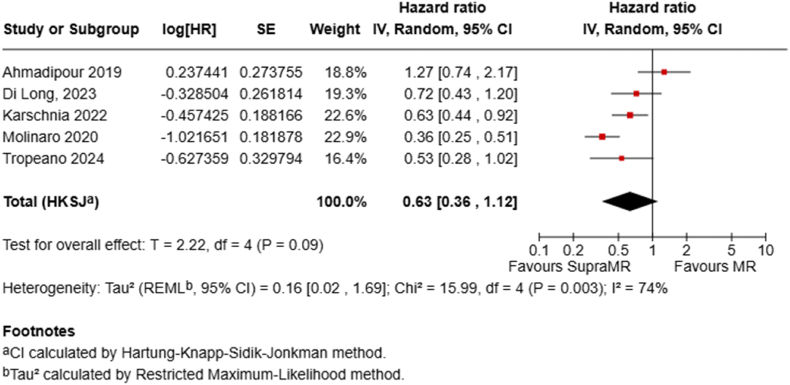
Forest plots depict HRs for SupraMR vs MR.

Five studies directly compared SupraMR with MR using time-to-event analyses. The pooled HR under a random-effects model was 0.63 (95 % CI 0.36–1.12; P = .09), indicating a non-significant 37 % reduction in the instantaneous risk of death with SupraMR at any given time point (Fig. 5). After removing the single outlier in a sensitivity analysis, we saw a significant decrease in HR to 0.53 (95 % CI, 0.32–0.88; P = .03) (Supplementary Fig. 6).

**Fig. 6:**
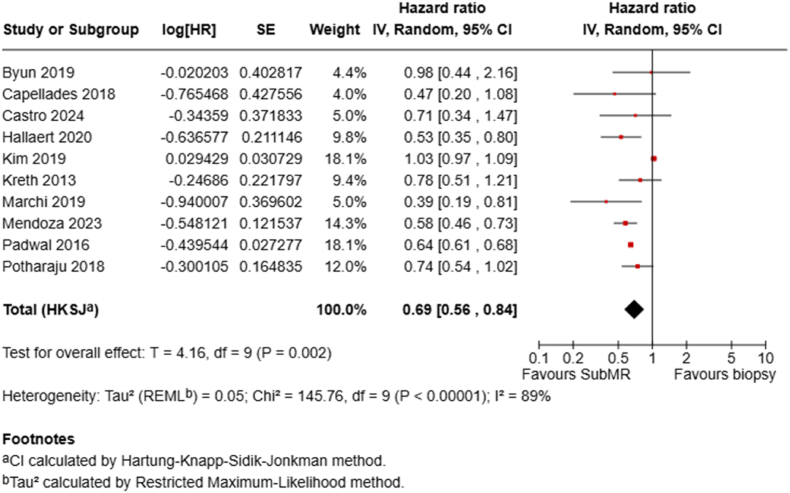
Forest plots depict HRs for MR vs subMR.

Twenty-one studies provided HRs for MR against subMR. The pooled HR was 0.69 (95 % CI, 0.63–0.74; P < .00001), demonstrating a highly significant 31 % reduction in mortality hazard for patients undergoing MR (Fig. 6).

**Fig. 7:**
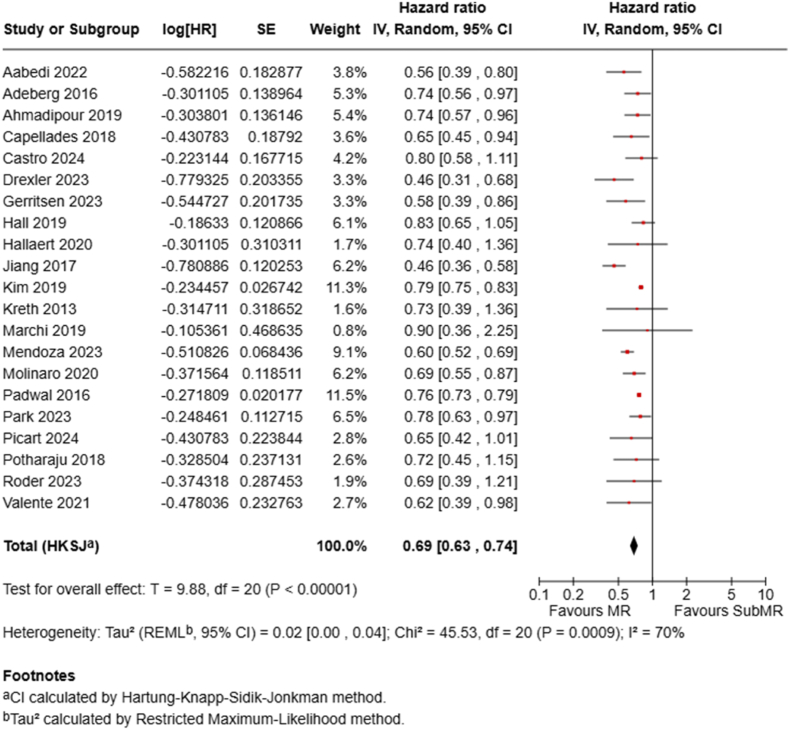
Forest plots depict HRs for subMR vs biopsy.

Ten studies compared subMR with biopsy alone using HR estimates. The random-effects pooled HR was 0.69 (95 % CI 0.56–0.84; P = .002), corresponding to a 31 % lower hazard of death after subMR compared to biopsy (Fig. 7).

### 3.7 Additional meta-analyses

We repeated the meta-analyses after removing both studies containing the SEER data (Kim et al., 2019, Padwal et al., 2016) as a sensitivity analysis to avoid distortion by these large studies, and again after removing the SEER study and the studies derived from composite RTOG data (Hall et al., 2019). In both instances, no significant change was found in the meta-analytic summary statistics for any comparison (Supplementary Table 2). Comparison of the relative RRs after removing these large datasets confirmed that no effect was found on the meta-analytic results (Supplementary Fig. 7). Additionally, we performed a meta-analysis at 1 and 2 years, comparing resection with biopsy (Supplementary Fig. 8). Across 12 studies comparing any surgical resection to biopsy alone, the pooled one-year OS analysis (left panel) yielded a RR of 0.61 (95 % CI, 0.50–0.75; P = .0003), indicating a 39 % relative reduction in mortality at one year among patients who underwent resection. At two years (right panel), the pooled RR was 0.84 (95 % CI, 0.77–0.91; P = .0008), corresponding to a 16 % relative mortality reduction with any resection compared to biopsy.

## 4 Discussion

This systematic review and meta-analysis aimed to clarify the impact of the EOR on OS, specifically in adult patients with newly diagnosed, IDH-wildtype GBM who were treated only with standard radiochemotherapy according to the EORTC 26981/22981 protocol. To our knowledge, this is the most comprehensive systematic review with quantitative meta-analysis to date. **By synthesizing data from 31 studies encompassing nearly 30.000 patients**, our findings demonstrate that greater EOR seems to be associated with a significant survival benefit.

### 4.1 Summary and interpretation of findings

Our analysis suggests a hierarchical survival benefit correlating with increasing EOR. Although median OS times between subMR and biopsy alone were not significantly different, a significantly reduced mortality risk at both one year (RR 0.76 (95 % CI 0.65–0.88) and two years (RR 0.91 (95 % CI 0.85–0.98) was seen for subMR, corresponding to a 31 % lower hazard of death (HR 0.69, 95 % CI 0.56–0.84). Moving further up the resection ladder, MR seemed to offer a notable survival advantage over subMR, with significantly lower mortality at one year (RR 0.59, 95 % CI 0.53–0.67) and two years (RR 0.82, 95 % CI 0.77–0.87), and a 31 % reduction in mortality hazard (HR 0.69, 95 % CI 0.63–0.74). Importantly, achieving SupraMR, defined as resection extending beyond the contrast-enhancing margins, seemed to confer an additional survival benefit compared to MR, particularly at two years (RR 0.70, 95 % CI 0.55–0.88). While the one-year mortality reduction for SupraMR vs MR reached borderline significance (RR 0.61, 95 % CI 0.38–0.99, P = .05), the HR comparison did not reach statistical significance (HR 0.63, 95 % CI 0.36–1.12, P = .09). This may be due to the limited number of studies (n = 5) in this specific comparison, which shows substantial heterogeneity. Furthermore, in a sensitivity analysis, the removal of the study by Ahmadipour et al., the only one that favored MR over SupraMR, resulted in a statistically significant effect in favor of SupraMR (HR 0.53, 95 % CI 0.32–0.88, P = .03).

The pooled survival estimates mainly show differences in the greater extent of resection groups: while mOS was 12.2 months with biopsy and 12.5 months with subMR, this increased to 17.5 months with MR and reached 28.2 months with SupraMR. Similarly, 2-year OS rates rose from approximately 19–22 % with biopsy/subMR to 29.1 % with MR and 51.8 % with SupraMR. These findings suggest that for IDH-wildtype GBM patients treated with standard concurrent chemoradiotherapy, maximizing safe resection, even beyond the contrast-enhancing tumor, may be associated with significantly prolonged survival. The mOS for subMR and biopsy groups are similar (12.5 months and 12.2 months, respectively), despite significantly better RR and HR for subMR compared to biopsy. It bears noting that one study that reports one of the lowest observed survival rates also contributes a very large part of the subMR population, This SEER analysis from Padwal et al. contributed 3498 of 10,925 patients (approximately 32 %) and significantly influences the median as well as the pooled 1- and 2-year survival rates. In sensitivity testing, excluding the three lowest-survival cohorts, which includes Padwal et al. (2016), raises the subMR 1-year OS from 49.1 % to 55.2 %, and similarly boosts the 2-year OS. This reflects a classic “large-study effect”: a single high-N, low-outcome cohort can dominate weighted analyses. Therefore, pooled survival metrics for subMR must be interpreted in light of the heavily weighted, lower-survival SEER dataset. Another potential explanation is that our “SubMR” category pools together patients who underwent subtotal resection with those who received only a partial resection. Survival outcomes of individuals in the partial resection subset typically tend to be significantly poorer than those of the subtotal‐resection cohort. When these two groups are analyzed as a single entity, the relatively worse results in the partial resection arm pull down the overall mOS, making the combined SubMR mOS appear lower than what we would observe if we looked at subtotal resections alone.

### 4.2 Comparison with recent literature

Our results both align with and refine the findings of previous large-scale analyses and recent studies focusing on IDH-wildtype GBM. The general principle that greater EOR improves survival in GBM has been established by earlier meta-analyses, such as Brown et al. (2016), and others, although these often included mixed IDH-status populations (Brown et al., 2016; Incekara et al., 2019; Revilla-Pacheco et al., 2021; Jackson et al., 2020). By focusing exclusively on IDH-wildtype patients treated since 2005, our study confirms that this survival association persists within this aggressive molecular subtype under the current standard of care with concomitant radiochemotherapy.

Several studies included in our meta-analysis provide context. For instance, Hallaert et al. (2020) specifically demonstrated a survival benefit for partial resection in comparison to biopsy in *MGMT-unmethylated* IDH-wildtype GBM, supporting our pooled finding that subMR seems to be superior to biopsy. Similarly, Byun et al. (2019) used propensity score matching and found improved survival with partial resection compared to biopsy in primary GBM, which again aligns with our results. By aggregating data from multiple cohorts, our meta-analysis reinforces and extends the findings of these individual studies.

The benefit of MR over subMR observed in our analysis (RR 0.59 at 1 year, RR 0.82 at 2 years) is consistent with the meta-analysis by Revilla-Pacheco et al. (2021) and Brown et al. (2016), which also reported superior survival for MR versus subMR in GBM patients. However, some of the studies included in the analysis by Brown et al. did not report whether IDH-status was specified, nor whether patients received the EORTC 26981/22981 protocol. In our analysis, the IDHwt status and treatment with the concomitant radiochemotherapy were specific selection criteria for inclusion.

The emerging concept of SupraMR yielding further benefit is supported by several recent studies included in our analysis, such as Molinaro et al. (2020), Di et al. (2023), Pessina et al. (2017), and Tropeano et al. (2024). Among these, Molinaro et al. notably showed that resection of both contrast-enhancing and non-contrast-enhancing (FLAIR) tumor components was associated with improved survival, particularly in IDH-wildtype patients. Our pooled analysis suggesting improved 2-year survival for SupraMR versus MR (RR 0.70) corroborates these findings, suggesting that targeting the infiltrative tumor margin beyond visible contrast enhancement may be a critical determinant of long-term outcomes. The recently proposed RANO-resect classification by Karschnia et al. (2022), also included in the meta-analysis, aimed to standardize EOR definitions, including non-contrast-enhancing tumor components. Its validation supports the prognostic relevance of achieving maximal resection of all identifiable tumor components.

However, the pursuit of maximal resection must be balanced against functional outcomes. Aabedi et al. (2022) highlighted that the relative survival benefit of maximal EOR might be diminished in patients experiencing significant postoperative neurological impairment. Gerritsen et al. (2023) also investigated the impact of maximal EOR on postoperative deficits and functioning, emphasizing the need to consider patient subgroups and potential trade-offs. While our meta-analysis focused on survival, the importance of preserving neurological function alongside maximizing EOR remains paramount in clinical decision-making (Gerritsen et al., 2021).

## 5 Study limitations

The present meta-analysis draws its data from a heterogeneous body of literature, the majority of which comprises retrospective observational studies, including retrospective cohorts and population-based reports, supplemented by only one paper with post-hoc exploratory analysis from an RCT (i.e., Hall et al. (2019), which pools data from NRG Oncology RTOG 0525 and 0825). This uneven evidence base introduces potential selection bias, as patients selected for more aggressive interventions, such as MR or SupraMR, may have had inherently favorable prognostic characteristics (e.g., higher performance status or tumors in less eloquent brain regions), which can confound comparative outcome assessments.

Geographic and institutional variability further complicates interpretation. While large, multicenter, and national registry studies enhance external validity, their integration with single-center case series or smaller cohort studies can amplify heterogeneity and limit the generalizability of any single-pooled estimate across diverse clinical settings. This diversity is compounded by wide variations in study objectives, from assessing the effects of EOR to evaluating drug efficacy or profiling molecular prognostic factors, as well as by differences in patient demographics, comorbidity profiles, and treatment timelines.

Several methodological inconsistencies quickly became evident when comparing the definitions of key variables and outcome measures. Definitions of subMR, MR, and SupraMR varied across studies, and comparator groups were often defined differently, resulting in divergent standards for reporting survival endpoints. Although random effects models were applied to account for statistical heterogeneity, these techniques cannot fully mitigate the underlying clinical and methodological diversity embedded in the source data.

A more substantial limitation of our analysis lies in the inconsistent and often incomplete reporting of critical molecular and clinical covariates across the included studies. The ideal analysis would account for all major prognostic factors to isolate the independent effect of the surgical intervention. However, we found that crucial data points were frequently undocumented or entirely absent.

For instance, MGMT promoter methylation status, a primary predictor of response to temozolomide chemotherapy, was one of the most significant omissions. Without this molecular data, it is difficult to uncover the benefits of resection from the inherent biological sensitivity of a patient's tumor to standard-of-care adjuvant therapy. Similarly, baseline functional status, typically measured by the Karnofsky Performance Status (KPS) was not uniformly available. Patients with a higher KPS are known to have better outcomes regardless of treatment, introducing a significant potential for confounding if one treatment group systematically included patients with better performance scores. Other vital parameters like preoperative tumor volume and the interval between surgery and the initiation of adjuvant therapy were also reported sporadically. We chose to exclude papers investigating the EOR specifically in elderly patients. This choice was made to not implement additional bias since higher age has been shown to negatively influence OS (Gorlia et al., 2008).

This lack of standardized data collection restricted our ability to perform a comprehensive multivariate adjustment. Consequently, we were unable to control for these powerful confounding variables statistically, and the pooled effect estimates may be biased. There is a risk that our results are skewed toward interventions that simply appear more favorable due to being applied to patient populations with intrinsically better prognoses, a benefit derived from incomplete covariate control rather than the intervention itself. This uncertainty tempers the strength of our conclusions and underscores the need for more rigorous data reporting in future clinical research.

Moreover, the evolving landscape of glioma diagnostics and surgical definitions over the course of the 2013–2024 publication window introduces temporal heterogeneity. Revisions to the WHO classification system in 2016 and 2021 redefined molecular subtype thresholds. To ensure a maximally homogenous study population, we established stringent inclusion criteria. Studies were included only if a minimum of 90 % of the enrolled patients had histopathologically confirmed IDH-wildtype glioblastoma. For cases where the IDH status was unknown, histopathological confirmation of glioblastoma was mandatory. This approach was adopted because, as reported by Eckel-Passow et al., 10 % of glioblastomas with typical histological features may harbor an IDH mutation (Eckel-Passow et al., 2015). Consequently, we estimate that the potential inclusion of IDH-mutant gliomas in our patient cohort is negligible, accounting for less than 1 % of the total population.

Meanwhile, the emerging recognition of non-contrast-enhancing tumor components (i.e., FLAIR-positive tissue) has altered the conceptualization of EOR. Earlier studies, which relied solely on contrast-enhancing margins, may therefore not align with contemporary surgical frameworks, further complicating cross-study comparisons.

The limitations summarized here represent the gap in current evidence in GBM surgery: most studies are retrospective in nature, have varying definitions of resection classes and harbour potential selection bias. To bridge this gap, uniform data collection, aligned with current WHO molecular criteria and standardized resection frameworks, such as RANO-Resect are warranted. These should be applied in prospective controlled and/or randomized studies, and this meta-analysis aims to provide the best knowledge base currently available to form hypotheses for future prospective randomized studies.

## 6 Clinical implications and future directions

Despite these limitations, our findings provide new, synthesized evidence in support of pursuing maximal safe resection in newly diagnosed IDH-wildtype GBM patients receiving postoperative concurrent radiochemotherapy. The data suggest a dose-response relationship, where greater EOR correlates with increasingly better survival outcomes, potentially extending to resection beyond contrast enhancement (SupraMR). Clinicians can use the pooled 1- and 2-year survival estimates (Table 1) to counsel patients, emphasizing that while a biopsy confirms the diagnosis, subMR offers a modest survival benefit, and MR provides a substantial advantage. SupraMR may offer the most significant potential for prolonged survival, albeit with an increased risk of functional impairment depending on tumor localization.

The decision regarding the optimal EOR must always be individualized, carefully weighing the potential survival gains against the risks of neurological deficits, particularly for tumors in eloquent areas. Advanced techniques, such as intraoperative mapping, 5-ALA fluorescence, and intraoperative MRI, are crucial tools for maximizing safe resection.

Future research should prioritize prospective, multicenter studies, that are either randomized or, when randomization is not deemed feasible, use a rigorously selected external control, and well-designed registry-based cohorts that employ standardized definitions for EOR (e.g., RANO-resect), uniform molecular characterization (including MGMT status and other relevant markers), and have consistent collection of functional outcome data alongside survival data. Such studies are essential for establishing the optimal EOR threshold, clarifying the role and safety of SupraMR across different tumor localizations and molecular subtypes within IDH-wildtype GBM, and better quantifying the trade-offs between EOR and quality of life.

## 7 Conclusion

This meta-analysis supports the association between increased EOR and improved OS in patients with newly diagnosed IDH-wildtype GBM treated with the EORTC 26981/22981 protocol. A hierarchical benefit was observed, with MR superior to subMR, and SupraMR potentially introducing further advantage over MR, particularly after 2 years. While maximizing safe resection should be the goal, the potential benefits must be carefully balanced against the risk of neurological impairment on a case-by-case basis. Further prospective and preferentially randomized research is warranted to provide definitive answers as to what constitutes the optimal surgical strategy for GBM patients. The balanced summary of survival data for each resection class provided in this review can serve as a foundation for effect estimation and sample size calculations in future trials.

## Declaration of competing interest

This research did not receive any specific grant from funding agencies in the public, commercial, or not-for-profit sectors.

The authors declare no conflicts of interest.
