# Supplementary material for: Extent of resection and its association with overall survival in newly diagnosed IDH wildtype glioblastoma treated with concomitant radiochemotherapy: a systematic review and meta-analysis
Source: Brain Spine. 2025 Nov 21;5:105867. doi: 10.1016/j.bas.2025.105867 (PMC12731770; doi:10.1016/j.bas.2025.105867)
Supplement: Multimedia component 1 [file mmc1.docx]

**Supplementary files**

| **Study** | **Study type** | **Total NOS Score** | **Selection (4)** | **Comparability (2)** | **Outcome (3)** |
| --- | --- | --- | --- | --- | --- |
| **Aabedi et al. (2022)** | **Retrospective cohort study** | **9** | **4** | **2** | **3** |
| **Adeberg et al. (2016)** | **Retrospective cohort study** | **7** | **4** | **1** | **2** |
| **Ahmadipour et al. (2019)** | **Retrospective cohort study** | **7** | **4** | **1** | **2** |
| **Amelot et al. (2017)** | **Retrospective cohort study** | **7** | **3** | **2** | **2** |
| **Byun et al. (2019)** | **Retrospective cohort study with propensity matching** | **8** | **4** | **2** | **2** |
| **Capellades et al. (2018)** | **Retrospective cohort study** | **9** | **4** | **2** | **3** |
| **Castro et al. (2024)** | **Retrospective cohort study** | **6** | **3** | **1** | **2** |
| **Di L et al. (2023)** | **Retrospective cohort study** | **9** | **4** | **2** | **3** |
| **Drexler et al. (2023)** | **Retrospective cohort study** | **9** | **4** | **2** | **3** |
| **Gerritsen et al. (2023)** | **Retrospective cohort study with propensity matching** | **9** | **4** | **2** | **3** |
| **Hall et al. (2019)** | **Posthoc exploratory analysis from RCTs** | **8** | **4** | **2** | **2** |
| **Hallaert et al. (2020)** | **Retrospective cohort study** | **9** | **4** | **2** | **3** |
| **Jiang et al. (2017)** | **Retrospective cohort study with propensity score matching** | **8** | **4** | **2** | **2** |
| **Karschnia et al. (2022)** | **Retrospective cohort study** | **9** | **4** | **2** | **3** |
| **Kim et al. (2019)** | **Retrospective cohort study** | **8** | **4** | **2** | **2** |
| **Kreth et al. (2013)** | **Prospective cohort study** | **8** | **4** | **2** | **2** |
| **Marchi et al. (2019)** | **Retrospective cohort study** | **7** | **3** | **2** | **2** |
| **Mendoza Mireles et al. (2023)** | **Retrospective cohort study** | **9** | **4** | **2** | **3** |
| **Molinaro et al. (2020)** | **Retrospective cohort study** | **9** | **4** | **2** | **3** |
| **Padwal et al. (2016)** | **Retrospective cohort study** | **6** | **3** | **1** | **2** |
| **Park et al. (2023)** | **Retrospective cohort study** | **9** | **4** | **2** | **3** |
| **Pessina et al. (2017)** | **Retrospective cohort study** | **8** | **4** | **2** | **2** |
| **Picart et al. (2024)** | **Prospective Randomized single blinded study** | **9** | **4** | **2** | **3** |
| **Potharaju et al. (2018)** | **Retrospective cohort study** | **6** | **3** | **1** | **2** |
| **Roder et al. (2023)** | **Prospective non-randomized controled trial** | **9** | **4** | **2** | **3** |
| **Salvati et al. (2020)** | **Retrospective cohort study** | **8** | **4** | **2** | **2** |
| **Tropeano et al. (2024)** | **Retrospective cohort study** | **9** | **4** | **2** | **3** |
| **Valente Aguiar et al. (2021)** | **Retrospective cohort study** | **6** | **3** | **1** | **2** |
| **Villanueva-Meyer et al. (2017)** | **Retrospective cohort study** | **8** | **4** | **2** | **2** |
| **Yang et al. (2022)** | **Retrospective cohort study** | **7** | **3** | **2** | **2** |
| **Yoo et al. (2022)** | **Retrospective cohort study** | **9** | **4** | **2** | **3** |

Supplementary Table 1: NOS scores for each of the included studies

**
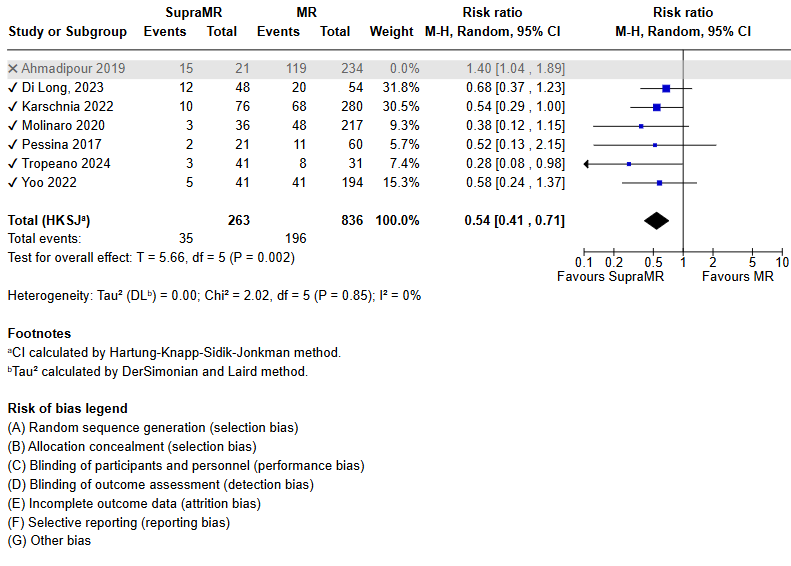
**

Supplementary Figure 1: RR at 1y after removal of the single outlier in the sensitivity analysis


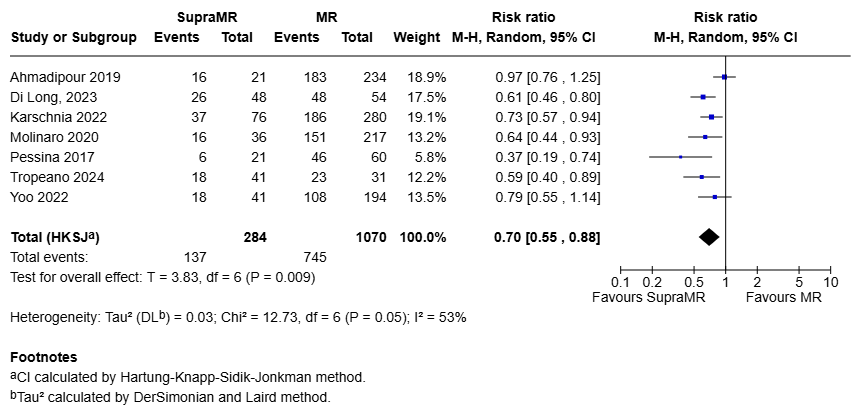


Supplementary Figure 2: Forest plots depict RRs at 2 years for SupraMR vs MR.

**
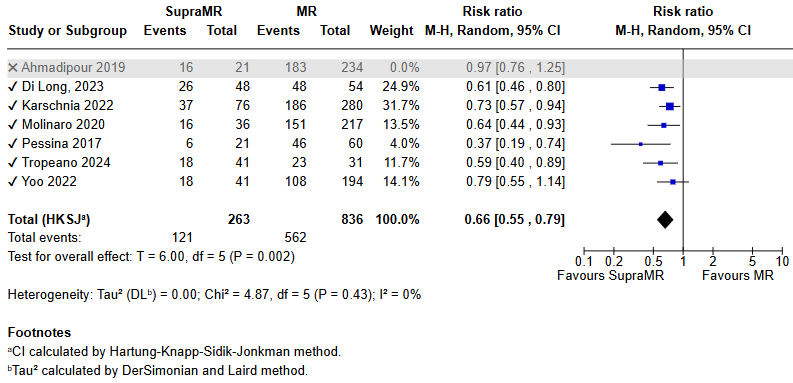
**

Supplementary Figure 3: RR at 2y after removal of the single outlier in the sensitivity analysis


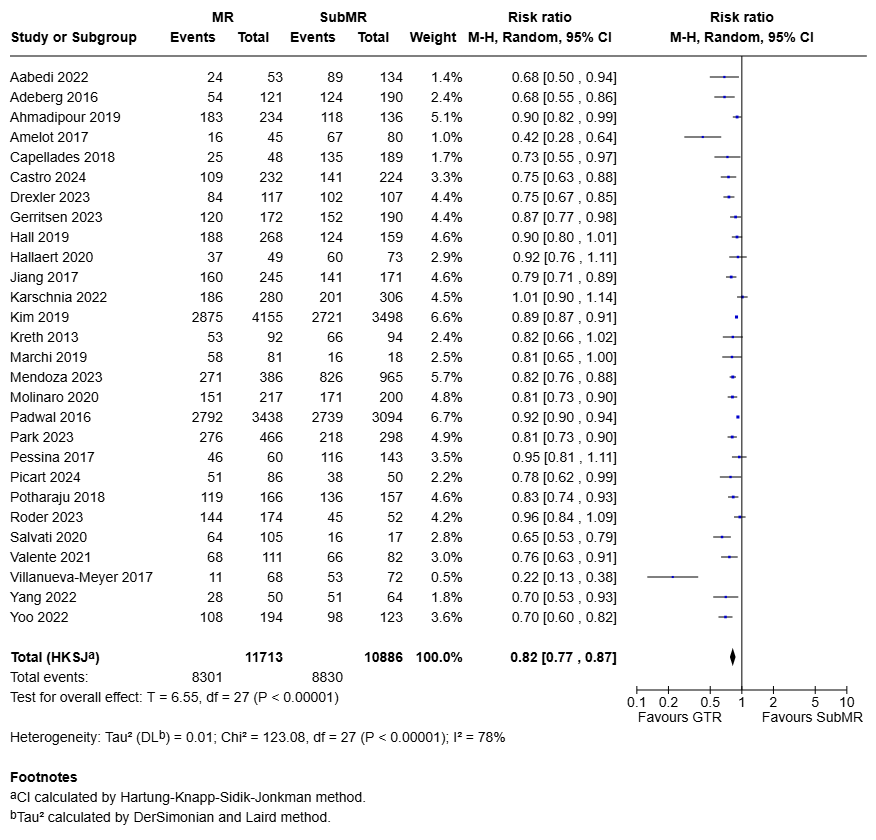


Supplementary Figure 4: Forest plots depict RRs at 2 years for MR vs subMR.


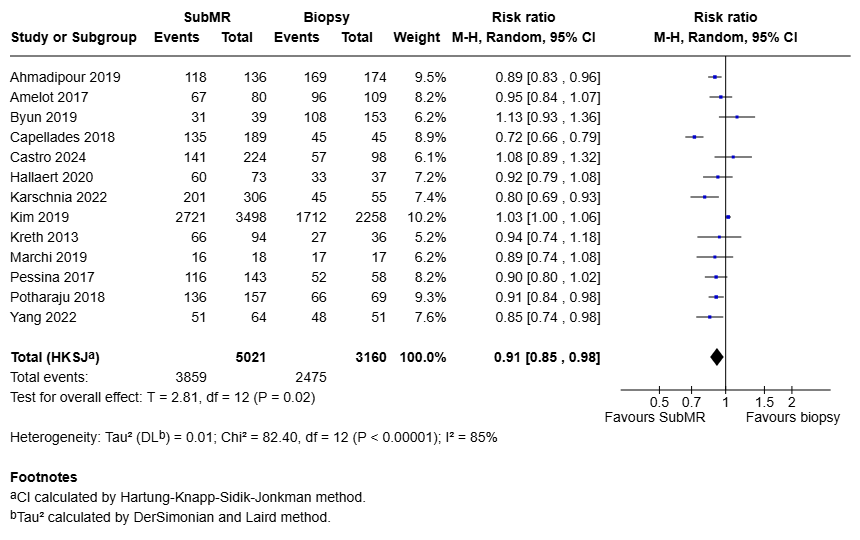


Supplementary Figure 5: Forest plots depict RRs at 2 years for subMR vs biopsy


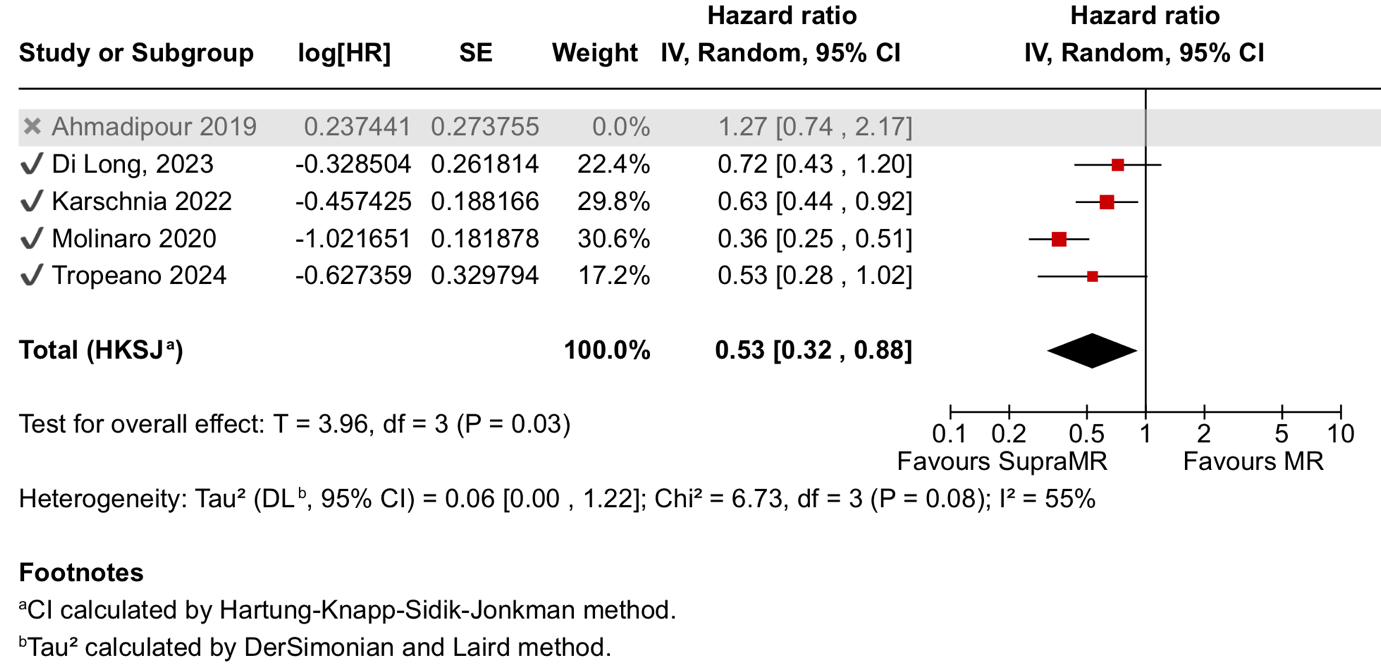


Supplementary Figure 6: HR for supraMR after removal of the single outlier in the sensitivity analysis

| **Comparison** | **RR overall** | **RR without SEER** | **RR without SEER & RTOG** |
| --- | --- | --- | --- |
| 1 yr RR mortality SupraTR vs GTR | 0.61 [0.38–0.99] p=0.05 | *N/A* | *N/A* |
| 2 yr RR mortality SupraTR vs GTR | 0.70 [0.55-0.88]  p =0.009 | *N/A* | *N/A* |
| 1 yr RR mortality GTR vs subMR | 0.59 [0.53–0.67] p<0.00001 | 0.56 [0.50–0.64] p<0.00001 | 0.55 [0.49–0.63] p<0.00001 |
| *2 yr RR mortality GTR vs subMR* | 0.82 [0.77-0.87]  p<0.00001 | 0.80 [0.74–0.86] p<0.00001 | 0.79 [0.73–0.86] p<0.0001 |
| 1 yr RR mortality subMR vs Bx | 0.76 [0.65–0.88] p=0.001 | 0.72 [0.63–0.82] p=0.0002 | 0.72 [0.63–0.82] p=0.0002 |
| 2 yr RR mortality subMR vs Bx | 0.91 [0.85–0.98] p=0.02 | 0.90 [0.83-0.97]  p=0.008 | 0.90 [0.83-0.97]  p=0.008 |
| 1 yr RR mortality Any vs Bx | 0.61 [0.51-0.74]  p=0.0001 | 0.58 [0.49-0.70]  p<0.0001 | 0.58 [0.49-0.70]  p<0.0001 |
| 2 yr RR mortality Any vs Bx | 0.83 [0.77 , 0.90] p=0.0003 | 0.82 [0.76-0.88]  p=0.0001 | 0.82 [0.76-0.88]  p=0.0001 |

**Supplementary Table 2**: Results of additional meta-analyses. Studies using SEER data were Kim 2019 and Padwal 2016. Hall 2019 contained pooled data from RTOG studies.

**
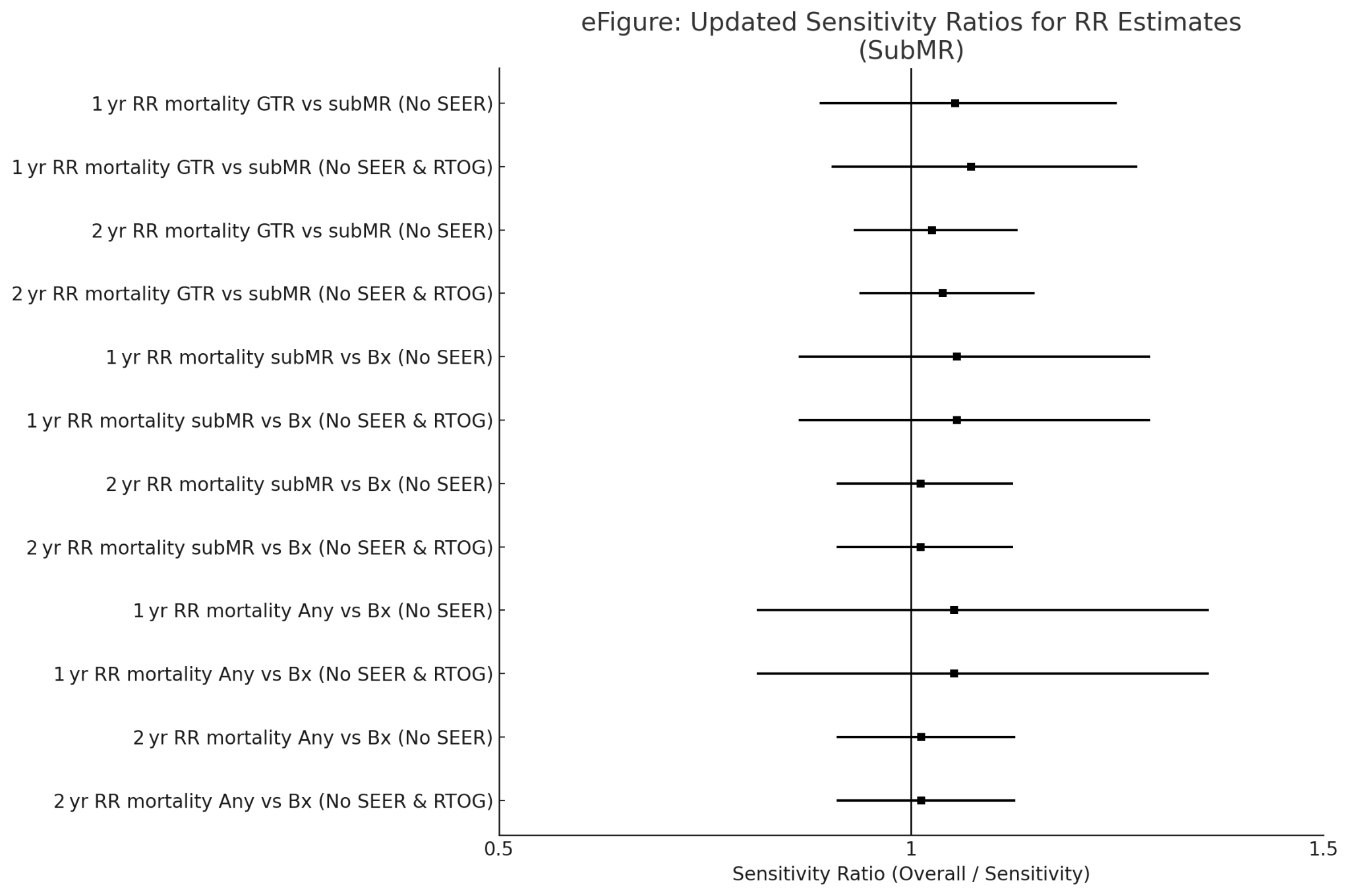
**

**Supplementary figure 7:** The relative risk ratios for mortality at 1- and 2-year time points were compared between the original meta-analysis and these sensitivity analyses (Supplementary Table 1). These comparisons revealed minimal changes in the overall meta-analytic estimates, suggesting that the inclusion of SEER and RTOG data did not significantly alter the findings. Therefore, these datasets were deemed representative of the broader population and the existing body of literature.


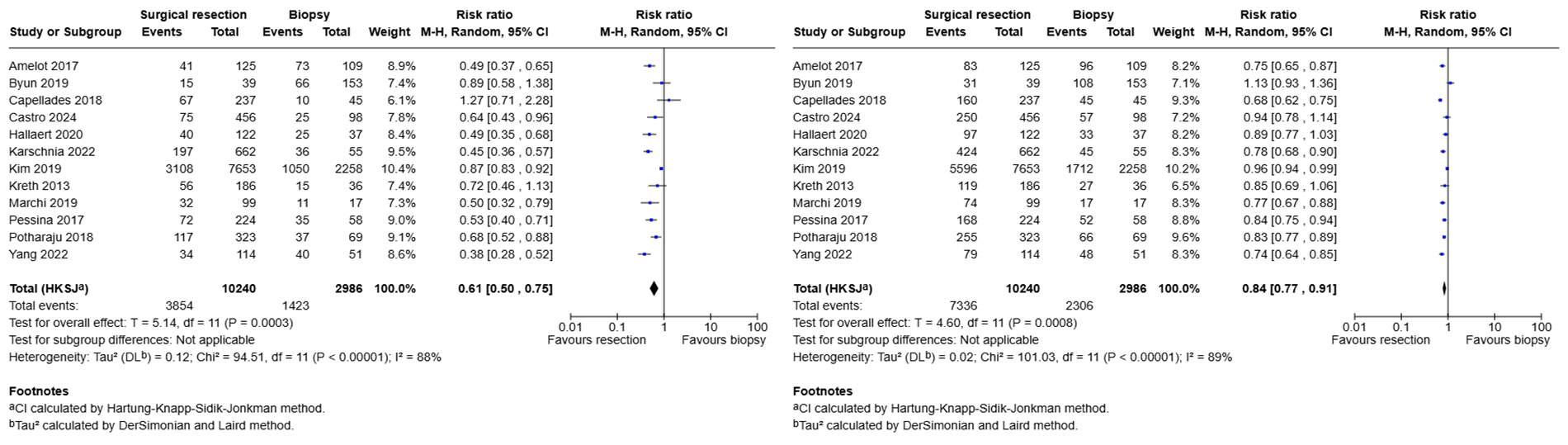


**Supplementary figure 8:** RR for 1 (left) and 2 year mortality (right) comparing any resection vs biopsy
